# Potential Use of Vivascope for Real-Time Histological Evaluation in Endoscopic Laryngeal Surgery

**DOI:** 10.3390/jpm13081252

**Published:** 2023-08-12

**Authors:** Luigi De Benedetto, Antonio Moffa, Peter Baptista, Simone Di Giovanni, Lucrezia Giorgi, Martina Verri, Chiara Taffon, Anna Crescenzi, Manuele Casale

**Affiliations:** 1 Unit of Integrated Therapies in Otolaryngology, Fondazione Policlinico Universitario Campus Bio-Medico, Via Alvaro del Portillo, 00128 Rome, Italym.casale@policlinicocampus.it (M.C.); 2 School of Medicine, Università Campus Bio-Medico di Roma, 00128 Rome, Italy; 3 Department of Otorhinolaryngology, Clinica Universidad de Navarra, 31008 Pamplona, Spain; 4 ENT Department, Al Zahra Private Hospital Dubai, Dubai 23614, United Arab Emirates; 5 Unit of Measurements and Biomedical Instrumentation, Università Campus Bio-Medico di Roma, 00128 Rome, Italy; 6 Unit of Endocrine Organs and Neuromuscolar Pathology, Fondazione Policlinico Universitario Campus Bio-Medico, 00128 Rome, Italy; 7 Pathology Unit, Fondazione Policlinico Universitario Campus Bio-Medico, 00128 Rome, Italy

**Keywords:** laryngeal dysplasia, artificial intelligence, endoscopic laryngeal surgery, confocal laser scanning microscopy

## Abstract

We aimed to assess the feasibility of using confocal laser scanning microscopy (CLSM) for the real-time ex vivo examination of histological samples of laryngeal lesions and to evaluate the correlation between CLSM and definitive histological results. This preliminary study included eight consecutive patients with “suspected” laryngeal lesions who were candidates for endoscopic laryngeal surgery. The obtained samples were evaluated using CLSM and classified as “inadequate” or “adequate” (high- and low-grade dysplasia, in situ and invasive carcinoma, positive surgical margin, and inflammatory outbreaks). CLSM showed the macro image in all cases and generated a digital version. All the samples were defined as adequate during CLSM and confirmed at histopathology: low-grade dysplasia (*n* = 5), low- and high-grade dysplasia (*n* = 2), and high-grade dysplasia (*n* = 1). Four samples had an involved resection margin, and three samples revealed the presence of inflammatory outbreaks. CLSM can be applied to larynx pathology with excellent agreement with final histological results.

## 1. Introduction

The diagnosis and treatment of premalignant and malignant laryngeal lesions are still significant challenges in the field of otolaryngology. It is well known that incisional biopsies (single or multiple) do not have an accuracy comparable to a definitive histologic diagnosis because they are not representative of the entire lesion. Usually, this kind of biopsy can cause undue damage and fibrosis of the multilayer structure of the vocal cord [1,2]. Therefore, all sophisticated preoperative and intraoperative diagnostic techniques would become ineffective or, at least, greatly hindered after laryngeal surgery [3,4]. Moreover, intraoperative pathology consultations for the lesion’s margins are unreliable as they are not representative of the state of the entire resection surface. Excisional biopsy, which allows the complete removal of the lesion with a regrowth of healthy tissue, is currently the gold standard for distinguishing suspicious from non-suspicious lesions [5]. However, this approach can sometimes increase the risk of positive surgical margins and require more extensive surgery to be completely radical [6]. If the margins are not disease-free, it is appropriate to consider revision laryngeal surgery, radiotherapy, or open surgery [1]. Patients with positive surgical margins can be scheduled for revision surgery 30–40 days after the first surgical procedure to ensure complete wound healing and a better evaluation of the neoglottis without excessive risk of bleeding [7]. Patients who have already undergone surgery present specific histopathology problems due to the presence of granulation tissue and an abundant inflammatory infiltrate and will require new general anesthesia [8,9]. Still, the problem of improving the boundaries of safety margins remains unresolved. New ex vivo optical imaging techniques, such as full-field optical coherence tomography, multiphoton microscopy, and confocal laser scanning microscopy (CLSM), have recently appeared. In particular, CLSM is a new optical technology intended for fast microscopic digital imaging directly from fresh, unfixed biological specimens without the need for slide preparation; this technique is called instant digital pathology [10]. This technique is exciting in the diagnostic field as it allows a rapid evaluation of surgical samples and small tissue fragments [11]. Unlike currently available techniques for immediate histological evaluation, CLSM produces an image of the macroscopic and microscopic features of the specimens, requiring minimal tissue preparation without causing any damage, distortion, or loss of tissue. Recent literature shows studies evaluating the usefulness of CLSM in the skin obtained from Mohs microscopic surgery for the recognition of non-melanocytic tumors [12], in urologic surgery for the intraoperative margin status assessment [13], and in the setting of pancreatic endoscopic ultrasound-guided fine needle biopsy (EUS-FNB) [14]. However, there are no data on CLSM use in laryngeal lesions. No information on sample preparation and/or protocols for managing laryngeal cellular material has been standardized and published. This study aims to assess the feasibility of applying the Vivascope 2500M-G4 (MAVIG GmbH Vivascope Systems) for real-time ex vivo examination of histological samples and micro-fragments of solid laryngeal lesions by defining the appropriate protocol for sample preparation and evaluation.

## 2. Materials and Methods

This is a single-center prospective study conducted on consecutive patients with "suspected" laryngeal lesions who are candidates for endoscopic laryngeal surgery at the Unit of Integrated Therapies in Otolaryngology of Fondazione Policlinico Universitario Campus Bio-Medico of Rome (Italy) from May 2021 to July 2022. This study conforms to the ethical guidelines of the 1975 Declaration of Helsinki. Written informed consent was obtained from all patients before their endoscopic examination procedures. 

The lesions were selected according to the following criteria:

- Inclusion criteria: leukoplakia, erythroplakia, erythroleucoplakia, or other mucosal lesions of one or both vocal cords that have not regressed after appropriate medical therapy.
- Exclusion criteria: submucosal lesions, lesions greater than 2 mm thick, surface extension greater than 2 cm, significant extension above or subglottic, refusal of informed consent, and age less than 18 years.

### 2.1. Tissue Collection and Preparation 

**Figure 1:**
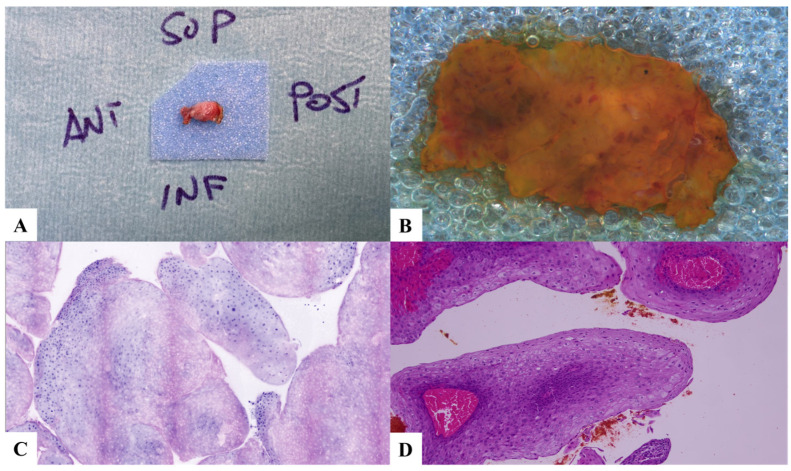
Case 1. Low-grade dysplasia and papillomatosis. (**A**) Laryngeal specimen placed in a biocassette according to the orientation scheme. (**B**) Laryngeal specimen details. (**C**) Vivascope. (**D**) Definitive histological examination. SUP: superior; POST: posterior; INF: inferior; ANT: anterior.

**Figure 2:**
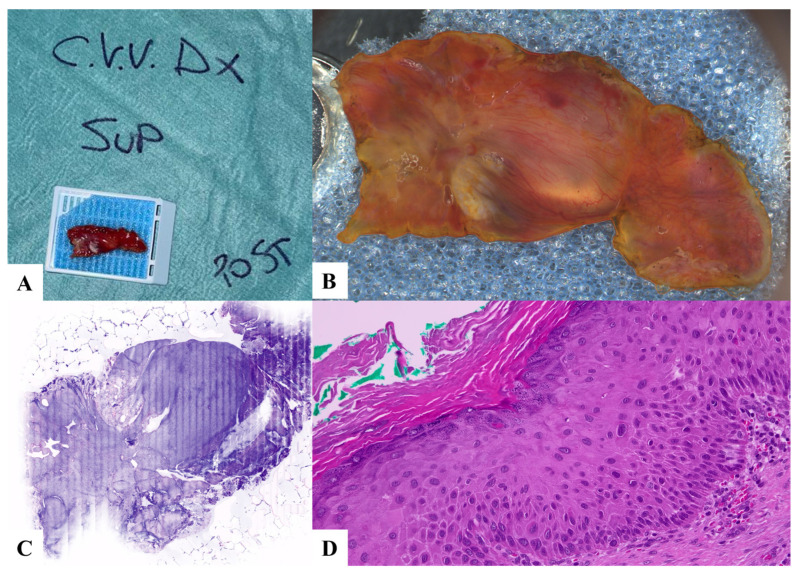
Case 2. High- and low-grade dysplasia, hyperkeratosis. (**A**) Laryngeal specimen placed in a biocassette according to the orientation scheme. (**B**) Laryngeal specimen details. (**C**) Vivascope. (**D**) Definitive histological examination. C.V.V DX: right true vocal cord; SUP: superior; POST: posterior.

**Figure 3:**
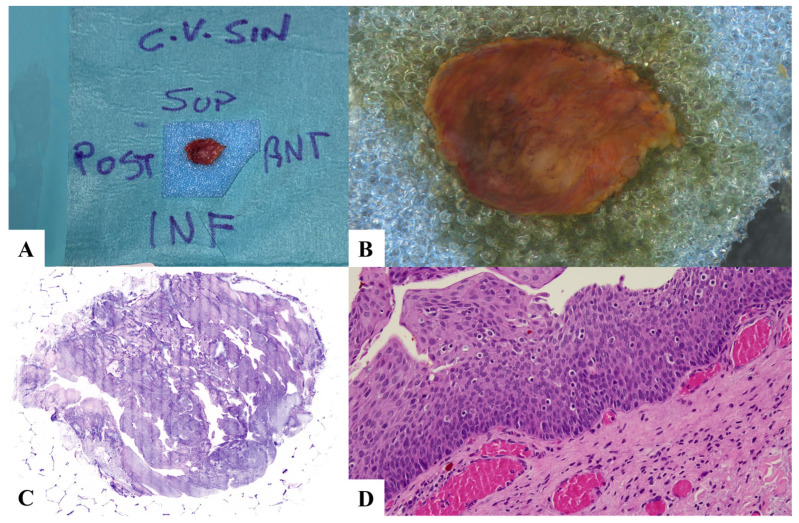
Case 3. low-grade dysplasia and lymphocyte exocytosis. (**A**) Laryngeal specimen placed in a biocassette according to the orientation scheme. (**B**) Laryngeal specimen details. (**C**) Vivascope. (**D**) Definitive histological examination. C.V. SIN: left vocal cord; SUP: superior; POST: posterior; INF: inferior; ANT: anterior.

This step was performed immediately after the excision of the lesion with a cold instrument during endoscopic laryngeal surgery or with a CO_2_ laser. The fresh sample, not fixed, was stretched out and oriented on a biopsy sponge (BioOptica, Milan, Italy) and delivered to the Pathology Unit of Fondazione Policlinico Universitario Campus Bio-Medico of Rome. The material provided included a photo of the samples with the oriented margins (Figure 1A,B, Figure 2A,B and Figure 3A,B) to identify the surgical margins and the orientation of the lesion. The piece loaded on the biopsy sponge was immediately washed with some drops of sterile saline solution, and the excess liquid was drained. This step was followed by placing some drops of acridine-orange for 30 seconds at room temperature and a rapid washing with buffered saline solution. A product specialist from Vivascope trained the pathologists regarding sample preparation and device functions, such as fluorescent dye incubation time and laser beam intensity. Pathologists were already trained in conventional digital pathology and CLSM pathology. In addition, they managed digital images on the computer screen (Figure 1C,D, Figure 2C,D and Figure 3C,D). Two different pathologists independently performed the CLSM evaluation and the final diagnosis on paraffin sections. At the time of the last observation of paraffin sections, pathologists were blinded to the CLSM diagnostic hypothesis.

The resolution of the images obtained with Vivascope was 1024 × 1024 pixels (single field of view).

### 2.2. CLSM Assessment

The instrument used for this study is the Vivascope 2500M-G4 microscope (MAVIG GmbH Vivascope Systems). The Vivascope first provides a macroscopic image of the sample. This image allowed a coarse evaluation of the sample surface and was used to point to the confocal acquisition by choosing the appropriate focal plane to initiate the entire acquisition. After rapid confocal scanning of the sample, the Vivascope produces a digital microscopic image, transforming the acridine fluorescence into a blue/purple color for the nuclei and the reflectance signal into a red eosin color, thus, very similar to that of conventional histological staining with hematoxylin/eosin. The workflow from acridine staining to digital imaging requires less than 4 minutes. The digital image allows all the application tools of digital pathology, such as high-power field zoom (up to 500×), obtaining cellular and tissue structure measurements, and sharing images for remote viewing. The following classification will be used to describe the assessment via the laser scanning confocal microscope Vivascope 2500M-G4:

- Inadequate/Not diagnostic.
- Adequate, which includes all samples showing a satisfactory digital image.
- The following parameters shall be evaluated for all defined and appropriate samples:
- Presence or absence of invasive carcinoma and in situ carcinoma.
- Presence or absence of dysplasia; in the case of dysplasia, the distinction between high-grade dysplasia/low-grade dysplasia.
- Positive or negative surgical margin according to the orientation scheme.
- Presence or absence of inflammatory outbreaks.

### 2.3. Post-Vivascope Processing of the Sample

After the evaluation with Vivascope 2500M-G4, the samples were recovered from the object holder, placed in a biocassette, and fixed in neutral formalin buffered at 10%. The fixed sample was submitted for macroscopic sampling by marking each surgical margin with a different color. Standard processing involved dehydration with growing alcohols and treatment with organic solvents (e.g., xylene), allowing subsequent permeation by a wax (paraffin). The treated tissues were included to form paraffin "blocks" containing the tissue to be examined. The blocks were cut with a microtome into 3–4-micrometer-thick sections and colored according to H&E protocol. Two pathologists skilled in laryngeal pathology blindly evaluated the slides for the definitive diagnosis. The final histological diagnosis was reported per the WHO classification of head and neck tumors [15]. The pathological anatomy team interpreted the specimens (M.V., C.T., and A.C.).

### 2.4. Statistical Analysis

The agreement between the evaluation of the Vivascope 2500M-G4 and the final histological diagnosis on the same sample will be evaluated according to Cohen’s Kappa coefficient, with the null hypothesis of no agreement. A *p*-value < 0.05 was considered statistically significant. The data were analyzed using R version 4.1.2 (R Foundation for Statistical Computing, Vienna, Austria).

## 3. Results

### 3.1. Patients and Characteristics of Lesions

From May 2021 to July 2022, we recruited 10 patients with as many laryngeal injuries. At the end of our selection process, we included eight patients: five males and three females (average age: 67.7 ± 14.81) (Table 1).

Specifically, three lesions extended along the entire length of the vocal cord; three lesions were localized in the middle third of the vocal cord; and two were at the level of the anterior-middle third of the vocal cord. Six of these lesions were presented as leukoplakia, while the other two were lesions with a leuko-erythroplasia appearance. The average length of the lesions was 1.25 ± 0.65 mm, the average width was 0.75 ± 0.22 mm, and the thickness was 0.23 ± 0.08 mm (Table 1).

### 3.2. Evaluation Using Vivascope

In all cases, the Vivascope 2500M-G4 was used to create digital images of the fresh sample. Following the classification, all eight lesions were deemed fit for evaluation. The dysplasia grading system is as follows: low-grade dysplasia (*n* = 5), combined low- and high-grade dysplasia (*n* = 2), and high-grade dysplasia (*n* = 1). Additionally, four specimens had an involved resection margin. Inflammatory outbreaks were also observed in three of the eight analyzed samples.

### 3.3. Histological Evaluation of Definitive Paraffin Sections

As a result of the histopathological evaluation of the final sections of the same sample, all eight samples were defined as adequate, allowing for a diagnosis. The histological diagnoses of the included samples revealed the following results: low-grade dysplasia (*n* = 5), low- and high-grade dysplasia (*n* = 2), and high-grade dysplasia (*n* = 1). Four samples had an involved resection margin. Furthermore, the definitive histological examination revealed the presence of inflammatory outbreaks in three of the eight samples analyzed.

The final histopathological analysis of the laryngeal samples showed a high level of agreement on diagnosis between the Vivascope evaluation and the definitive histological examination for all parameters evaluated (Cohen coefficient k, 0.95; 95% CI, 0.89–1.01; *p* < 0.001).

## 4. Discussion

To the best of our knowledge, this preliminary report represents the first application of the Vivascope for laryngeal sample examination, suggesting that the histological results obtained via CLSM appear to be highly reliable in assessing the presence or absence of carcinoma and/or dysplasia (Table 1).

CLSM and specifically the Vivascope 2500M-G4 allow for a quick and fast microscopic examination of tissues, providing information on cell morphology and the relationship between epithelium and stroma. Vivascope has previously been used in other areas of medical diagnosis, with high sensitivity and specificity in detecting neoplastic cells [16]. Its usefulness has been evaluated in diagnosing non-melanoma skin tumors and on other types of surgical samples, including liver biopsy and prostatic tissue, showing how this technique can distinguish between neoplastic and non-neoplastic tissues [17,18,19]. The Vivascope 2500M-G4 offers several benefits. First, this tool allows for the possibility of quickly analyzing the unfixed fresh tissue, leading to considerable saving of time. Therefore, this would enable a “real-time” histological diagnosis of the lesion and its margins, allowing the surgeon to extend the resection, if necessary, during the same procedure. This way, the patient will not have to undergo revision surgery, avoiding the sacrifice of healthy tissue and the risks of new general anesthesia. It is necessary to highlight that the Vivascope examination does not alter histological results. Unlike the extemporaneous examination, characterized by distortions from the cryostat section, it keeps the tissue intact. CLSM could also offer the possibility of conducting a "remote diagnosis" by sharing digital images for remote counseling with pathologists worldwide and also provide the opportunity to build a library of digital images. This digital sharing can also be used to obtain a quick pathological consultation in a hospital where a pathology unit is unavailable, thus allowing for adequate evaluation through remote analysis. Vivascope images permit the application of neural networks for artificial intelligence algorithms. Because the fluorescence laser can be used alone, it is possible to obtain two-color photos similar to radiological ones, where various automatic recognition systems have already been implemented. 

According to a recent literature review, this technique was previously used for the ex vivo histological diagnosis of head and neck mucosal lesions. In particular, Just T. et al. [20] investigated and evaluated whether FCM could supply essential information for the surgeon prior to surgery for 9 patients with tongue cancer and 12 with head and neck cancer who underwent radiochemotherapy. The authors showed that dysplastic and cancerous lesions reveal significant differences compared to healthy oropharyngeal tissue. After RCT, several epithelial changes were found, such as keratosis, mild dysplasia, increased vascularization, increased cell edema, and necrosis [20].

Shavlokhova V. et al. [21] evaluated FCM for the first time for a fast-automated histological tissue examination of oral squamous cell carcinoma. The authors showed promising results in the automated classification of cancerous tissue from 20 patients, reaching a sensitivity of 0.47 and a specificity of 0.96 [21]. 

The same group analyzed the sensitivity and specificity of FCM for detecting oral leukoplakia by comparing confocal images with the gold-standard histopathology of 27 oral lesions. Leukoplakia was detected with an overall sensitivity of 96.3%, a specificity of 92.3%, a positive predictive value of 93%, and a negative predictive value of 96% [22]. 

The present study has some limitations. First, it is a single-center study conducted on a limited number of patients. Moreover, the pathologists received two years of training in using the Vivascope and specialized in diagnosing laryngeal lesions. This could potentially limit the reproducibility of the results. Furthermore, since Vivascope imaging is a new modality for histological examination, a learning curve and adequate preparation are necessary for pathologists to improve their knowledge of this technology and be more confident in interpreting the digital representation.

## 5. Conclusions

In conclusion, Vivascope 2500M-G4 represents a new instant digital diagnostic tool that has been successfully applied to larynx pathology with excellent agreement with the final histological results. It may offer the possibility of informing the patient of the diagnosis on the same day of the procedure, allowing for safe surgical resection margins.

## Figures and Tables

**Table 1:** Laryngeal lesions characteristics examined.

Id Patient	Male/Female (M: F)	Age (Years)	Anatomic Region	Dimension (cm)	Surgical Procedure	Evaluable with Vivascope (Yes/No)	Dysplasia and Grade	Surgical Margins	Inflammatory Outbreaks
01	F	63	Middle third of the left vocal cord	0.9 × 0.7 × 0.2	Cordectomy (type I) using cold steel	Yes	Low-grade dysplasia	Low-grade dysplasia on posterior margin (1/4)	Chronic inflammation
02	F	39	All the left vocal cord	1.1 × 0.6 × 0.1	Cordectomy (type I) using CO_2_ laser	Yes	Low-grade dysplasia	Free surgical margins	No inflammation
03	M	72	Middle third of the right vocal cord	1.5 × 1 × 0.3	Cordectomy (type I) using CO_2_ laser	Yes	Low-grade dysplasia	Free surgical margins	No inflammation
04	M	83	Third middle and anterior of the right vocal cord	2.7 × 1 × 0.3	Cordectomy (type II) using CO_2_ laser	Yes	Low and high-grade dysplasia	Free surgical margins	No inflammation
05	F	73	Third middle and anterior of the right vocal cord	0.6 × 0.5 × 0.2	Cordectomy (type I) using CO_2_ laser	Yes	High-grade dysplasia	All margins with low-grade dysplasia 6/6	Chronic inflammation
06	M	56	All the left vocal cord	1.4 × 1 × 0.2	Cordectomy (type II) using CO_2_ laser	Yes	Low-grade and high-grade dysplasia	Posterior margin (low-grade dysplasia) and inferior margin (high-grade dysplasia) 2/6	chronic inflammation
07	M	83	The middle third of the left vocal cord	1 × 0.7 × 0.3	Cordectomy (type I) using CO_2_ laser	Yes	Low-grade dysplasia	Anterior and inferior margin (low-grade dysplasia) 2/6	No inflammation
08	F	74	All the right vocal cord	0.8 × 0.5 × 0.3	Cordectomy (type I) using cold steel	Yes	Low-grade dysplasia	Free surgical margins	No inflammation

## Data Availability

The datasets collected and/or analyzed during the current study are available from the corresponding author upon reasonable request.
